# Our Contemporaries

**Published:** 1913-02

**Authors:** 


### OUR CONTEMPORARIES.

*The National Journal of Medicine and Surgery* begins Volume 107 with January, 1913. It was established 62 years ago (apparently starting with annual and changing to semi-annual volumes). We extend congratulations to one of the very few extant journals approaching our own age.

DR. DE LANCEY ROCHESTER of Buffalo contributes an excellent article on the Management of Pneumonia to the *Medical Council* of January, 1913.

THE ALBANY MEDICAL ANNALS, January, 1913, publishes an elaborate illustrated article on fracture of the femoral neck, by Dr. C. E. Ruth of Des Moines, too long for abstracting but deserving the attention of every surgeon.

THE N. Y. REAL ESTATE JOURNAL, January 15, 1913, contains an interesting article on Industrial Buffalo by Mr. Frederick H. Rice, Secretary of the Buffalo Real Estate Association.

